# Patient-level costing analysis of paediatric short bowel syndrome care in a specialist tertiary centre

**DOI:** 10.1007/s00383-022-05074-6

**Published:** 2022-02-24

**Authors:** Brendan C. Jones, Benjamin O’Sullivan, Sonal Parmar Amin, Susan Hill, Simon Eaton, Paolo De Coppi

**Affiliations:** 1 Developmental Biology and Cancer Research and Teaching Department, Great Ormond Street Institute of Child Health, University College London, London, UK; 2 Specialist Neonatal and Paediatric Surgery Unit, Great Ormond Street Hospital, London, UK; 3 Department of Paediatric Surgery, Bristol Royal Hospital for Children, Bristol, UK; 4 Finance Department, Great Ormond Street Hospital, London, UK; 5 Department of Gastroenterology, Great Ormond Street Hospital, London, UK

**Keywords:** Short bowel syndrome, Parenteral nutrition, Cost of care, Health economics

## Abstract

**Purpose:**

To undertake a pilot study estimating patient-level costs of care for paediatric short bowel syndrome (SBS) from the healthcare provider perspective.

**Methods:**

A pilot group of patients with anatomical SBS was selected at a single specialist tertiary centre in the United Kingdom. The Patient Level Information and Costing System (PLICS) was used to extract costing data for all hospital-based activities related to SBS, from the implementation of PLICS in 2016 to April 2021. Patient-specific and pooled data were reported descriptively in per patient-year terms.

**Results:**

Five patients had full PLICS data available for the 5-year study period and 2 patients had 4 years of data. The median cost for hospital care of SBS was £52,834 per patient-year (range £1804–£331,489). The key cost drivers were inpatient beds, pharmacy, and staffing costs, which made up > 60% of annual costs. In the first 3 years following index admission (*n* = 2), there was a steady decline in the annual cost of care to a level comparable with patients with established SBS.

**Conclusion:**

Patient-level cost of care analysis for SBS is feasible using PLICS. Hospital-related costs vary widely between and within individual patients over time. Key drivers of cost are related to complications of SBS.

## Introduction

Short bowel syndrome (SBS) is a clinical syndrome with several congenital and acquired causes defined by the lack of adequate functional intestinal epithelium to maintain hydration, nutritional balance, and support growth without additional parenteral nutrition. It is estimated to affect up to 1 in 25,000 patients in the developed world [1–3]. For those who survive the initial insult, SBS requires intensive inpatient, outpatient, and community-based care with input from multiple medical, surgical, nursing, and allied health specialities. Unless the patient achieves enteral autonomy through intestinal rehabilitation, there is currently no cure for SBS. Supportive treatments include parental nutrition (PN), intestinal lengthening procedures, and for those with severe disease and access to a specialist service, intestinal transplantation [4–6]. However, there are many complications of SBS, and currently available treatments are associated with significant side effects including, PN-associated liver failure, sepsis, replacement and loss of central venous access devices (CVAD), metabolic bone disease, transplant-associated immunosuppression, and ultimately death [5]. In children, SBS 5-year survival may be as low as 20%, while those undergoing small bowel transplantation have a 5-year survival of only 58% [7–9].

Investing in new treatments involves substantial R&D and translational costs, which should be carefully evaluated. Economic analysis is essential to informing population-level decision-making in healthcare management and policy. Novel technologies and interventions should demonstrate cost-effectiveness to justify the diversion of scarce resources from the current standard of care to a new therapy. For a specified cost, a new intervention should produce more health benefit than existing care. This requires that the cost of existing care is accurately known.

Most cost of care analyses for SBS focus on adult patients or mixed populations in which paediatric patients are not analysed separately [10]. The most robust assessment of comprehensive care in paediatric SBS was undertaken by the Teitelbaum group in Michigan, USA [11]. Their study assessed inpatient and outpatient costs of the first 5 years following SBS diagnosis over a period from 1992 to 2005. However, these data and others from similar studies were carried out predominantly from the 1990s to early 2000s and/or focus on the USA and Canada [12–16]. More recent studies tend to focus on one aspect of care, such as inpatient PN provision [17], central line-associated bloodstream infection (CLABSI) occurring in the context of home PN [18], include the highly variable cost of the index admission [19], or were performed as cost-effectiveness simulations [20].

The National Health Service (NHS) in the United Kingdom implemented the Patient Level Information and Costing System (PLICS) in 2016 [21]. PLICS provides a reflection of the cost of providing healthcare to patients by a given service provider in the NHS. Since its introduction, NHS Trusts have been mandated to use PLCIS data to report to the annual national cost collection each year, which helps to inform the NHS national tariffs that are set in the following 2 years. PLICS reports are also used to identify the profitability of services and engage clinical staff in-service evaluation and redesign to improve the efficiency and cost-effectiveness of patient care.

With this pilot study, we aimed to demonstrate the feasibility of the PLICS system to produce data estimating patient-specific costs of existing care of paediatric short bowel syndrome (SBS) from the perspective of an NHS trust. To avoid the marked variability related to the cost of care during the SBS-precipitating event [19], this study focuses on the estimate of costs of care on patients after discharge from the index admission.

## Methods

### Ethics approval statement

This study was undertaken following approval by the Great Ormond Street Hospital NHS Foundation Trust Quality and Safety Department as a service evaluation for the cost of care (approval number 3076). The terms of the evaluation were limited to the study of data available in the Trust PLICS system and data routinely recorded in the electronic medical record.

### Study design and patient selection

To leverage the newly implemented NHS England and NHS Improvement’s Costing Transformation Programme, we performed a retrospective analysis of patient-specific costs attributable to a single tertiary referral centre in London over a minimum time horizon of 4 years. This maximised use of the Patient Level Information and Costing System (PLICS) data collections, which were first piloted as PLICS Acute in 2016, with wider implementation from 2017 onwards [21]. PLICS methodology is described below.

Patient inclusion and exclusion criteria were determined by local multi-disciplinary expert consensus involving paediatric surgeons, specialist intestinal failure nurses, paediatric gastroenterologists, community nurses, and parenteral nutrition pharmacists. A sample of patients aged 0–16 years undergoing management for SBS at a UK tertiary referral centre were identified. Any cause of SBS was considered admissible, but intestinal failure due to functional aetiologies, rather than anatomically short bowel, were excluded. This was done to generate a dataset from a group of patients most likely to benefit from full-thickness intestinal tissue engineering, a new therapeutic option currently under active research [22].

Costs associated with index inpatient admissions were excluded, as it has been shown that some of the largest variations in the cost of care are introduced in the index admission [11, 19]. Furthermore, our aim was to assess the cost of care during the chronic or rehabilitation phase of SBS treatment, during which time the aim is to achieve enteral autonomy while minimising complications. Therefore, the start date for calculation of costs was 2 months after discharge from the index admission (i.e., the admission when the SBS-causing event occurred). Patients with significant co-morbidities were excluded to maintain the focus on costs of SBS-related care only. Inclusion and exclusion criteria are summarised in Table 1.

**Table 1:** Inclusion and exclusion criteria for this pilot study

Inclusion criteria	Exclusion criteria
Clinical diagnosis of SBS (all causes)	Significant non-SBS co-morbidity
Full 4 years PLICS costing data available	Home PN for < 12 months
Currently receiving home PN	Intestinal failure from functional cause
Minimum period of care for SBS following index admission: 1 year	

### Collection of resource use and unit cost data

As introduced above, we utilised the data prospectively collected through the Trust’s existing PLICS methodology. Inpatient and outpatient patient-specific healthcare resource use is captured within the Trust through normal clinical and administrative activity in the electronic patient reporting (EPR) system, Epic (Hyperspace Version August 2021, Epic Systems Corporation, USA). The data in these inpatient and outpatient activity data feeds are regularly pulled into the costing system by the PLICS team in the Trust’s Finance Department. The PLICS team then use additional resource utilisation data feeds, such as pathology test records, radiology test and procedure records, pharmacy drug records, and theatre procedure minutes data, to facilitate and verify patient-matched resource use, and then to track individual patient activity. Financial data which reflect the expenditure incurred from the running of the hospital, for example staffing costs and overheads (averaged per unit time), is then fed into PLICS to match appropriate proportions of these expenditures to individual patients. In this way, individual patient-level costs can be calculated for all inpatient and outpatient activity at the Trust.

PLICS data reflect the cost of delivering care and contribute to mandatory annual national reporting of costs by all UK trusts to NHS England. Data are prepared and audited against annual national clinical costing standards to assure quality [23]. Unit costs are applied within PLICS, according to local contractual and national tariffs, which are annually updated.

Using this methodology, PLICS data were obtained for each patient for full financial years over the study period between 2016 and 2021, which are all years that PLICS has been utilised in the NHS. Costs were categorised for each patient to identify clinically relevant key cost drivers. These categories included “beds and overheads”, “pathology”, “pharmacy and drugs”, “radiology”, “operating theatres and procedures”, “ward and outpatient staffing”. Operating theatres and procedure costs reflect the cost of the procedure itself, theatre and procedure room staff, and the cost of theatre or procedure room time. Home care providers for home PN are external to the Trust and engaged by the NHS through a confidential commercially sensitive agreement. Therefore, home care services did not fall under the terms of the study approval and are not included in this report.

### Data analysis and presentation

Primary outcome was average costs of care per patient per year in 2021 British Pounds Sterling (GBP). For reference, the current average GBP to Euro and US Dollars are as follows: £1 = €1.19 and £1 = $1.36. The preference for economic analysis is to present average costs, but the data used for this study show a clear non-parametric distribution. Therefore, for completeness, data are presented as both mean with 95% confidence interval and as median with range.

## Results

Seven patients were included in the pilot analysis. Demographic data for these patients are shown in Table 2. Five patients had full yearly PLICS data available over the 5-year study period and 2 patients had 4 years of data, resulting in a total of 33 patient years available for cost analysis. Total cost across all patients was £2,146,371, creating an average cost per patient per year of over £65,000 (95% CI [£39,171, £90,911).

**Table 2:** Demographic data for patients included in the pilot analysis

Patient no.	Age (years)	Sex	Aetiology of SBS	Date eligible for study inclusion
1	15	F	Antenatal volvulus and jejunal atresia	01/08/2006
2	14	M	Long-segment Hirschsprung’s disease	16/11/2008
3	10	M	Midgut volvulus	01/11/2011
4	7	F	Necrotising enterocolitis	01/06/2015
5	16	M	Bowel obstruction (internal hernia)	28/12/2016
6	5	M	Complex gastroschisis	06/07/2017
7	5	F	Complex gastroschisis	30/09/2017

**Fig. 1:**
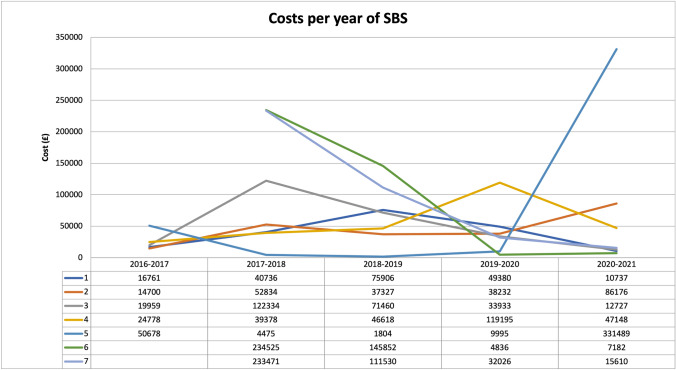
Patient-level cost of care for SBS treatment over the study period. Each of the patients (1–7) is represented by a different line to allow tracking of individual year-to-year changes

Significant variation in total yearly costs was evident across the period of study, driven by high cost-generating inpatient clinical events (Fig. 1) and resulting in a non-parametric distribution of costs. The median yearly patient-level healthcare cost was £52,834, but with a wide range from a minimum of £1804 to a maximum patient-year cost of £331,489 (Table 3).

**Table 3:** Median estimated cost per patient per year from PLICS data

Year	Median costs per patient (£)	Range per patient (£)
2016–2017	19,959	14,700–50,678
2017–2018	52,834	4475–234,525
2018–2019	71,460	1804–145,852
2019–2020	33,933	4836–119,195
2020–2021	73,009	7182–331,489
Whole study period	52,834	1804–331,489

Key cost drivers were the categories of inpatient “beds & overheads”, “pharmacy & drugs” and “ward & outpatient staffing” costs. These costs comprised a minimum of 60% of total patient care cost per annum and as high as 91% of the annual cost in 2018–2019. Table 4 summarises these main cost drivers as a proportion of pooled total cost per year. Remaining expenditure was comprised of costs associated with pathology, radiology, and use of theatres and procedure rooms, including proceduralist and other staffing costs.

**Table 4:** Key cost drivers as a percentage of total cost for annual pooled cost of all patients

Year	Total cost of care for all patients (£)	Bed, pharmacy and staffing costs (£)	Bed, pharmacy, and staffing costs percentage of total cost (%)
2016–2017	126,876	75,755	60
2017–2018	727,757	641,907	88
2018–2019	490,498	446,135	91
2019–2020	290,172	254,791	88
2020–2021	511,068	393,150	77
Total all years	2,146,371	1,811,738	84

As shown in Fig. 1, costs were relatively stable for most years for most patients. However, patient numbers 3, 4, and 5 all had one year (2017, 2019, or 2020, respectively) where costs were anomalously high compared to their other years of care. For patient 3, this was explained by two inpatient admissions: first for gastrostomy granuloma treatment and a feeding trial, and second for a laparoscopic-assisted gastrostomy revision, which was converted to an open procedure due to extensive intra-abdominal adhesions. In 2019, patient 4 had admissions for dislodgement of a peripherally inserted central catheter (PICC) and an episode of CLABSI requiring theexchange of CVAD. Finally, patient 5 had a nearly 4-month long admission with adhesive small bowel obstruction requiring laparotomy, which was subsequently complicated by 2 returns to theatre (early obstruction secondary to sealed perforation; early adhesive obstruction) and a prolonged ICU stay.

There were two patients for whom only 4 years of data were available (patient numbers 6 and 7). PLICS data were available for the entire period of their SBS diagnosis. As can be seen from Table 2 and Fig. 1, there is a steady downward trend in annual cost observed in the first 3 years following the diagnosis of SBS in these patients, reaching a level comparable to patients with more established SBS. Together, these pilot data suggest that patients with SBS are most likely to incur high hospital-related costs within the first 3 years after index admission, with these costs falling to a plateau beyond this time, unless a significant inpatient admission occurs.

## Discussion and conclusions

This pilot study provides an up-to-date patient-level estimate of healthcare costs from provider perspective for current standard care for paediatric SBS in the UK. A healthcare provider perspective is relevant to trust-level NHS decision-makers and healthcare commissioners. These data facilitate an initial prediction of long-term healthcare costs of the hospital-based component of paediatric SBS and serve as a proof-of-concept for the use of PLICS data to perform a more in-depth cost of care analyses for rare diseases.

Using a sample of patients from a tertiary paediatric hospital with a specialist multi-disciplinary intestinal failure service and patient-level costing data, we can estimate the median yearly cost for hospital-based SBS care at £52,834. However, we observed a wide range in annual costs both between and within patients, covering a range of £1804 to £331,489 per patient year. We found that inpatient bed, pharmacy (including PN), and staffing costs are the main drivers of total hospital-based cost per patient-year, which is consistent with the findings of a recent systematic review and emphasize the advantage of avoiding hospital admission in SBS patients while investing in home care [10].

To assess different stages of the disease, our pilot data include patients with both established SBS and patients soon after diagnosis. We observed stable annual hospital-related cost of care for established SBS patients, except when the occurrence of complications leads to hospital admission (Fig. 1). In newly diagnosed patients, there was a steady decline in annual cost over the available study years. This is consistent with the trend observed in a comprehensive American study of paediatric SBS cost of care, which found the highest cost in the first year following diagnosis (> US$500,000, pricing year 2005), with a rapid decline to a plateau of US$250,000–300,000 per patient year [11], suggesting our methodology is a valid approach in the UK context. Therefore, subsequent use of our study methodology and PLICS data will allow future studies to estimate the potential resource savings from the prevention of acute admissions. Ideally, such data can be used in building business cases for the development of programmes in the community to avoid complications requiring admission to hospital, as home PN has already been shown to be 60–76% less costly than in-hospital PN [10].

Due to the volume and breadth of cost-generating events in this patient group from intensive care admissions to home care visits, we recognise that our study estimates only the hospital-related costs of SBS care and deeper statistical analysis was limited by the pilot nature of our patient sample. PLICS is utilised by all NHS trusts to improve internal efficiency. However, its use limits the ability to present disaggregated resource use and unit costs. In addition, a healthcare provider perspective for analysis underestimates the substantial societal costs of SBS. A measure of indirect costs in this population and to their families would require prospective long-term follow-up through key stages of life including school, higher education, and occupation. These costs are outside the scope of this study but are extremely relevant when constructing future cost-utility analyses for new therapeutic options, especially as the last British study of this kind took place in 1996 and was limited to adult patients [24].

Furthermore, healthcare costs of paediatric SBS in the UK are incurred by the NHS through both tertiary centre hospital care and through contracting to home care providers. We have not included home care data in this analysis, as stated in the methods. Therefore, the cost data provided represent a significant underestimation of the wider costs to the NHS. The current NHS National Framework Agreement for the supply of home parenteral nutrition and intravenous fluid support for patients with intestinal failure details the cost of delivery of home PN for adults and children, including installation of devices required in the home (for example, a medicines refrigerator), home delivery, PN products, pumps and stands, nursing, and ancillaries costs [25]. While access to the details of this commercially sensitive agreement cannot be disclosed outside of the NHS, the approximate minimum cost of home care can be estimated at £40,000 per patient-year from the agreement. Although this is likely an underestimation of the current true cost of home care in the UK, it is substantially less than published costs for the USA (at least US$87,932 per patient-year over first 5 years post-diagnosis, in 2005 prices) [11] or Canada (up to CAD$320,369 in the first year following discharge from index admission, in 2014 prices) [16]. In a European context, previously reported costs for paediatric home PN range from €46,000 (pricing year 2006) [19] to €230,000 (pricing year 2012) [20] per patient-year. These differences likely reflect the different health care system payer models between the three jurisdictions, as well as the time point post-diagnosis of included patients. However, it is interesting to note that Spencer et al. [11] observed a steady increase in the cost of home care over time, which was related to the persistently high cost of home PN and the need for additional therapies for complications, including antimicrobial therapy. Adding the average hospital-related cost calculated in this study to the current estimated minimum home care cost under the current NHS National Framework Agreement, the average cost to the NHS of around £100,000 per patient-year, ranging from an approximate minimum £42,000 to approximate maximum of £370,000, for patients with SBS.

Given that home care costs are relatively fixed in the United Kingdom, it can be argued from this pilot study that avoidance of inpatient admissions during intestinal rehabilitation is economically most important in minimising cost of care. This is supported in a European context by a Belgian study of intestinal failure patients that, for children, home care costs were relatively stable over time and the majority of the cost of care was attributable to treatment of complications and the underlying disease. By contrast, the dominant cost for adults in the same study was the provision of home PN [26]. While achievement of intestinal autonomy is the ultimate goal, there is evidence for those with access to intestinal transplantation that it may be more cost effective than home PN as little as two years after transplant [27]. However, 5-year survival rate of 58% and the continued requirement for immunosuppression demonstrate the substantial ongoing economic and health costs associated with intestinal transplantation [28], justify the continued search for curative SBS treatments, including cell-based and tissue engineering approaches [29–31].

In conclusion, our study shows that patient-level cost of care analysis is feasible, even for rare conditions like SBS. Hospital-related costs vary widely from year to year between and within individual patients, with significant outlier costs related to complications of SBS and its care, including bowel obstruction, CLABSI, feeding ostomy formation, and ICU admission. Avoiding these clinical events that drive high costs is definitively done by achievement of enteral autonomy. This justifies the investment in intestinal rehabilitation programs and the research into the development of a tissue engineering solution to SBS.
